# Diagnostic radiography and adult acute myeloid leukaemia: an interview and medical chart review study

**DOI:** 10.1038/bjc.2011.114

**Published:** 2011-04-26

**Authors:** J M Pogoda, P W Nichols, R K Ross, D O Stram, D C Thomas, S Preston-Martin

**Affiliations:** 1 Department of Preventive Medicine, Keck School of Medicine of the University of Southern California, Los Angeles CA 90089, USA; 2 Department of Pathology, Keck School of Medicine of the University of Southern California, Los Angeles CA 90089, USA

**Keywords:** acute myeloid leukaemia, case-control study, ionising radiation, radiography, diagnostic imaging

## Abstract

**Background:**

Aetiology of acute myeloid leukaemia (AML) is not well understood, perhaps because of its distinct subtypes. High-dose ionising radiation is a known risk factor, but less is known about risk from low-dose exposure such as from diagnostic radiography.

**Methods:**

Subjects were 412 matched case-control pairs. Ten-year subject histories of diagnostic radiography were based on interview and medical records.

**Results:**

There was no convincing association between AML risk and ionising radiation exposure from diagnostic imaging procedures, either for AML overall or for any AML subtype.

**Conclusion:**

The association between diagnostic radiography and AML risk remains uncertain.

Aetiology of acute myeloid leukaemia (AML) is not well understood, perhaps related to the fact that AML consists of morphologically and genetically distinct subtypes. High-dose ionising radiation is one of the known causes (Darby *et al*, 1987; Preston *et al*, 1987), but less is known about risk from lower-dose exposures, such as those received from diagnostic procedures.

The French–American–British (FAB) classification in use at the time of our study divided AML into seven subtypes based on morphological and cytochemical criteria (Bennett *et al*, 1976, 1985a, 1985b). It seems plausible that specific exposures may correlate with specific mutations in either stem or progenitor white blood cells due to their vulnerability to particular carcinogens at different stages of cellular growth and differentiation (Sandler *et al*, 1993). The resulting phenotypic changes that constitute the different FAB subtypes may also have unique risk factor profiles.

We report results from a large, population-based case-control study of AML in Los Angeles County, California in which data on diagnostic imaging procedures were collected from both interview and medical records. Because we were able to determine FAB subtype on almost 90% of cases, it was feasible to evaluate whether AML risk from diagnostic radiography may be specific to certain AML subtypes.

## Materials and methods

The study protocol was approved by the University of Southern California (USC) Institutional Review Board. Study design and methodology have been previously described (Pogoda *et al*, 2002). New cases of adult-onset AML (ICDO codes 9861, 9864, 9866, 9867, and 9891) diagnosed in Los Angeles County from January 1987 through June 1994 were identified by the USC Cancer Surveillance Program (CSP), a population-based SEER cancer registry. Other inclusion criteria included age between 30 and 69 years, the ability to speak English or Spanish, and U.S. residence during the previous 15 years. Neighbourhood controls were matched to cases by birth year (±5 years), ethnicity, and sex according to a previously established protocol (Preston-Martin *et al*, 1980).

Using the FAB classification scheme that was standard at the time the study was conducted, cases were FAB-subtyped by review of pathology reports (96%) or slides (4%) by one of us, an experienced hematopathologist (PWN). For cases with FAB subtype not specified on the pathology report or for whom diagnostic information was otherwise incomplete, available peripheral blood and bone marrow slides were reviewed to verify the original AML diagnosis and to establish the FAB subtype.

Interviews were conducted from 1987 to 1997. Specific details of procedures used to ascertain histories of diagnostic radiography and to estimate dose have been published previously (Preston-Martin and Pogoda, 2003). Respondents were systematically queried on a specific list of imaging procedures and were also asked to provide contact information for all providers they saw in the 10 years before diagnosis, whether or not the provider ordered or delivered diagnostic imaging procedures; these providers were asked to provide details on all diagnostic imaging procedures undergone by the subject during the 10 years of interest. Providers were also asked for names of any other providers who had treated the patient, and these additional providers were also contacted and asked to provide the same information. A comparison of exposure data from personal interviews v*s* medical records has been published previously (Pogoda and Preston-Martin, 2002). Assignment of likely radiation dose to the bone marrow for each procedure was accomplished by literature review and radiology expert consultation (Preston-Martin and Pogoda, 2003). In brief, we searched the literature for estimates of radiation dose to the active red bone marrow for each procedure reported by our respondents and used procedure-specific medians for our analysis. For procedures for which we were unable to find literature-based estimates, we consulted with radiologists to arrive at reasonable estimates to use for analysis. We used the conversion equation 1 Gy=100 rad to convert all estimates to mGy.

Radiographic exposure was analysed as number of frequently reported procedures (per medical records) and as total dose (mGy) received. Categorical analyses were done using logistic regression conditioned on matched pairs (Breslow and Day, 1980) and adjusted for socioeconomic status (SES) (Hollingshead, 1957); cut points were chosen *a priori*. Trend tests and multivariable analyses of dose by age at exposure were done using continuous exposure variables in logistic models. Population attributable risk (PAR) was calculated using the continuous exposure variable (Bruzzi *et al*, 1985). As the PAR is a monotonic transformation of the risk coefficient, its confidence interval (CI) was derived directly from the risk coefficient likelihood-based CI. Primary analyses excluded matched pairs in which the case or control had missing exposure data and was limited to exposure in the 2–10 years before diagnosis. A secondary analysis retained pairs with missing data and used the missing indicator method (Huberman and Langholz, 1999). Analyses were done using SAS v9 (SAS Institute, Inc., Cary, NC, USA) and Epicure v2.11 (Hirosoft International Corp., Seattle, WA, USA). Tests were two-sided with 0.05 significance levels.

## Results

The CSP identified 725 cases of AML diagnosed January 1987 to June 1994 who met inclusion criteria. Of these, 188 (26%) were unavailable for interview with no available proxy, 31 (4%) were not contacted as advised by their physicians, and 20 (3%) were lost to follow-up. Therefore, 67% (486 out of 725) of eligible cases were invited and 412 agreed to participate, or 57% (412 out of 725) of those originally identified (85% (412 out of 486) of those contacted. Proxies were used for 49% (201 out of 412) of cases. Median length of time from AML diagnosis to interview was 7 months for personal and 14 months for proxy interviews. Median number of months between case and control interview was 8.

Distributions for demographic variables and FAB subtype are shown in Table 1. FAB subtype for proxy-interviewed cases was more likely to be M0, M5, or missing than for those personally interviewed. Cases and controls were similar on age and sex. Despite matching by neighbourhood of residence, cases were somewhat more likely to be Latino and to have lower SES. Of 51 cases (12%) with unknown FAB subtypes, most (82%) were due to inadequate pathology materials being available. FAB subtypes of cases who were eligible but did not participate in the study were unknown.

Reasons for case-control pair exclusion in analyses were case diagnosis of refractory anaemia with excess blasts (RAEB) or in transformation (RAEB-T), also known as myelodysplastic syndrome (*n*=15), prior radiation therapy (*n*=38), and prior chemotherapy (*n*=18) Also, 64 pairs were excluded from analysis of medical record data because of data unavailability. Therefore, 344 matched pairs (83% of pairs interviewed) were included in analysis of interview data and 279 pairs (68% of pairs interviewed) in analysis of medical record data. Among those included, the average number of health care providers identified in interviews was similar for cases and controls (4.0 and 3.7, respectively), as was the average number of additional providers identified by contacted providers (2.0 for cases and 1.4 for controls).

The diagnostic imaging procedures most frequently recorded in subjects' medical records, used in the ‘number of procedures’ analysis, are shown in Table 2. The most common as well as the lowest-dose procedure was chest x-ray, with nearly 50% of all respondents having had at least one during the period of interest, whereas nuclear medicine scan had the highest estimated dose. A natural cut point in the distribution of dose was 1 mGy, and this was used to discriminate between ‘low-dose’ and ‘high-dose’ procedures in analyses.

Table 3 shows risk estimates for total number of high- and low-dose frequently reported diagnostic imaging procedures. For subtype M4, number of high-dose procedures appeared positively associated with AML risk (*P* trend=0.03 based on interview data, Table 3), as did total dose from radiographic procedures (Table 4). However, this relationship appears to result from M4 controls being unusual in that they had relatively few high-dose procedures and low total exposure compared with all controls combined. There were no discrepancies among controls by FAB subtype in distributions of factors (age, sex, ethnicity, and SES) that might be associated with access to or willingness to seek medical care (data not shown). There were no other apparent dose-response relationships suggesting increased risk, either for all pairs combined or for specific FAB subtypes. PAR based on medical records was 5% (95% CI:−3%, 11%) for all pairs combined. In multivariable analyses of cumulative dose during three different periods of age at exposure – 0–39, 40–54, and 55+ years – no particular age period was associated with significantly increased risk. Analyses based on medical records that retained all matched pairs but included an indicator covariate for missing exposure data, produced results similar to the primary analyses (data not shown).

## Discussion

We observed no apparent relationship between radiation exposure from diagnostic imaging procedures and adult AML risk, confirming the lack of association reported from earlier epidemiological studies (Linos *et al*, 1980; Boice Jr *et al*, 1991; Zheng *et al*, 1993; Yuasa *et al*, 1997). In fact, there have been no prior reports showing convincing evidence of such an association. Stewart *et al* (1962) reported an association between trunk x-rays and myeloid and monocytic leukaemia, but 11 years later attributed it to diagnostic procedures related to the leukaemia disease process. A more recent study from Japan suggested that an association between AML risk and conditions that may lead to increased x-ray exposure (e.g., bone fracture) links AML risk to x-ray exposure (Wakabayashi *et al*, 1994), but data on x-ray exposure were not collected and thus this hypothesis could not be tested directly.

The lack of evidence from epidemiological studies of an association between adult AML and diagnostic radiography supports the BEIR VII model for leukaemia, which predicts a relative risk of 1.017 per 0.01 Sv (equivalent to 10 mGy) exposure for a male over 30-years old, 10 years after exposure (Board on Radiation Effects Research, 2006). This effect size would be very difficult to detect in an epidemiological study of diagnostic radiography, given the high levels of exposure that would be required, the exposure typically received from these types of procedures (10 mGy=about 200 chest x-rays), and the rarity of AML.

It is worth mentioning that the BEIR VII analysis excluded leukaemia cases that occurred during the first 5 years after atomic bomb exposure, yet several studies of medically exposed cohorts have reported highest relative risks in the 1–5 years after exposure (Board on Radiation Effects Research, 2006). Thus, it is possible that limitations in our study prevented us from detecting increased AML risk from recent exposure. For example, a relatively small proportion (57%) of eligible cases were able to participate in the study; this is an inherent problem in any epidemiological study of AML, which must rely on the case-control design due to the low incidence of disease. Because median survival after AML diagnosis is measured in months v*s* years, and because our median time to interview was 7 months, non-participation in our study was largely due to death (and lack of an available proxy). If non-participating cases had more exposure in the few years just before diagnosis – for example, if radiation exposure causes a more rapidly fatal form of AML – our study would have missed this potential causal effect.

Exposure misclassification can result in biased estimates of disease risk. All indicators we were able to examine (e.g., comparison of interview with medical record data) suggested that misclassification in our study was either non-differential, which tends to bias risk estimates towards the null, or was present in a way that would bias towards the null; for example, proxy respondents underreporting to a greater degree than non-proxies (Pogoda and Preston-Martin, 2002).

Estimates of US radiographic doses are uncertain because not all relevant details, such as number of retakes, were considered (Preston-Martin and Pogoda, 2003). Failure to consider retake rate consistently underestimates per-procedure dose to the patient. We conducted a telephone survey of Los Angeles County facilities that provide diagnostic radiography (*n*=174) to ascertain retake rates for common procedures and found that mean retake rate for the most common procedures ranged from 5.5% (range=1.2–18.2%) for upper gastrointestinal series to 24.7% (range=1–100%) for routine chest examinations. Several common high-dose procedures had relatively high average retake rates, for example, lumbosacral spine examinations (mean 16.2%, range=4–53%). Our dose estimates are also likely to consistently underestimate actual doses delivered because they are derived from dosimetry surveys from the US and other Western countries, all of which assume use of ideal radiographic conditions (Preston-Martin and Pogoda, 2003).

In summary, our study has not provided convincing evidence supporting an association between diagnostic radiography and adult AML risk. Although such an association would not be expected based on the BEIR VII report, there remains uncertainty regarding the first 5 years after exposure, and there are enough limitations to a case-control study such as ours that it is not possible to conclude that such an increased risk does not exist. Further, it is important to note that radiation exposure from diagnostic imaging procedures increases with improved image quality due to improved equipment (Smith-Bindman, 2010) which, coupled with the dramatic increased use of imaging by medical practitioners since our study was conducted (Hillman and Goldsmith, 2010), suggests that our subjects were exposed to lower doses of radiation from diagnostic procedures than they would have been in the current healthcare environment.

## Figures and Tables

**Table 1:** Distribution of study subjects by sex, age, ethnicity, SES, and FAB subtype

**Characteristic**	**# Cases (%)**	**# Controls (%)**
*Sex*		
Male	234 (57)	234 (57)
Female	178 (43)	178 (43)
		
*Age (years)*		
25–39	57 (14)	57 (14)
40–49	67 (16)	74 (18)
50–59	109 (26)	104 (25)
60–75	179 (43)	177 (43)
		
*Ethnicity*		
Non-Hispanic white	306 (74)	334 (81)
Hispanic	65 (16)	45 (11)
African American	37 (9)	31 (8)
Other	4 (1)	2 (0.5)
		
*SES* a		
Low	217 (53)	193 (47)
High	195 (47)	218 (53)
Unknown	0 (0)	1 (0.002)
		
*FAB Subtype*		
M0	7 (2)^b^	
M1	70 (19)	
M2	116 (32)	
M3	34 (9)	
M4	89 (25)	
M4E	4 (1)	
M5	17 (5)	
M5a	3 (1)	
M5b	6 (2)	
Other^c^	15 (4)	
Unknown	51	

Abbreviations: FAB=French–American–British; RAEB=refractory anaemia with excess blasts; RAEB-T=refractory anaemia with excess blasts in transformation; SES=socioeconomic status.

a Based on education and occupation according to the Hollingshead Social Index (Hollingshead, 1957): low>51, high⩽51.

b Percentages=% of cases with non-missing FAB.

c Includes RAEB (*n*=4) and RAEB-T (*n*=11).

**Table 2:** Most frequently recorded diagnostic imaging procedures in medical records

**Procedure**	**Estimated dose^a^ (mGy)**	**# Subjects with exam recorded (*n*=716)^b^**	**Total # exams recorded**	**Avg # exams per subject among those with exam**
Nuclear med scan, high dose^c^	15.83	47	60	1.3
GI tract CT	14.56	13	20	1.5
Coronary angiogram	12.67	28	41	1.5
GI series^d^	5.05	107	158	1.5
Spine CT	4.13	20	33	1.7
Head/neck CT scan	3.00	35	55	1.6
KUB	2.14	51	61	1.2
GI tract x-ray	1.54	34	51	1.5
Spine x-ray	1.41	140	226	1.6
Hip/pelvis x-ray	0.57	42	68	1.6
Rib/sternum x-ray	0.49	26	39	1.5
Fluoroscopy, extremity	0.39	13	18	1.4
Shoulder x-ray	0.37	36	46	1.3
Nuclear med scan, low dose^e^	0.36	23	28	1.2
Head/neck x-ray	0.16	43	59	1.4
Mammogram	0.07	112	253	2.3
Chest x-ray	0.05	331	987	3.0
Extremity x-ray	0.00	158	400	2.5
Abdominal ultrasound	0.00	25	30	1.2
Hip/pelvis ultrasound	0.00	20	32	1.6
Hepatobiliary ultrasound	0.00	17	20	1.2

Abbreviations: Avg=average; CT=computed tomography; GI=gastrointestinal tract; KUB=kidneys/ureters/bladder.

a Dose to active bone marrow, equal to the median dose among all procedures in the given category. In contrast, procedure-specific dose estimates were used for analysis. For reference purposes, the average annual background exposure in the US is 3 mGy (Board on Radiation Effects Research, 2006).

b Of 824 study participants, 38 case-control pairs were excluded due to prior radiation treatment of either the case or control (or both), and an additional 18 pairs were excluded due to prior chemotherapy.

c At least 2 mGy (based on specific scan performed).

d Includes both upper and lower GI; for analysis, doses for upper and lower GI were estimated separately.

e Range=0.0001–0.72 mGy (based on specific scan performed).

**Table 3:** Risk analysis of number of most frequently reported diagnostic imaging procedures in the 2–10 years before diagnosis, adjusted for SES^a^

		**Data from medical records**										**Data from interview**									
		**High dose^b^**					**Low dose^b^**					**High dose^b^**					**Low dose^b^**				
**FAB group**	**# Exams**	**# Ca**	**# Co**	**OR**	**(95% CI)**	***P* trend^c^**	**# Ca**	**# Co**	**OR**	**(95% CI)**	***P* trend^c^**	**# Ca**	**# Co**	**OR**	**(95% CI)**	***P* trend^c^**	**# Ca**	**# Co**	**OR**	**(95% CI)**	***P* trend^c^**
All^d^	0	162	146	1.0		0.74	95	88	1.0		0.11	212	199	1.0		0.54	104	80	1.0		0.41
	1–4	98	120	0.7	(0.5, 1.0)		136	116	1.0	(0.7, 1.5)		117	140	0.8	(0.6, 1.1)		164	168	0.7	(0.5, 1.1)	
	⩾5	19	13	1.2	(0.6, 2.4)		48	75	0.5	(0.3, 0.9)		15	5	2.7	(1.0, 7.5)		76	96	0.6	(0.4, 1.0)	
																					
M1	0	34	31	1.0		0.21	19	13	1.0		0.03	41	41	1.0		0.80	24	14	1.0		0.52
	1–4	19	19	0.9	(0.4, 2.0)		28	25	0.7	(0.3, 1.8)		26	26	1.0	(0.5, 2.0)		25	34	0.4	(0.2, 1.0)	
	⩾5	2	5	0.4	(0.1, 2.1)		8	17	0.3	(0.1, 1.0)		0	0	—	—		18	19	0.5	(0.2, 1.4)	
																					
M2	0	42	45	1.0		0.44	25	26	1.0		0.20	65	55	1.0		0.57	29	25	1.0		0.19
	1–4	32	31	1.0	(0.5, 2.1)		42	28	2.0	(0.9, 4.3)		29	41	0.6	(0.3, 1.2)		52	45	1.2	(0.6, 2.3)	
	⩾5	5	3	1.6	(0.4, 6.8)		12	25	0.4	(0.1, 1.2)		5	3	1.4	(0.3, 6.2)		18	29	0.7	(0.3, 1.6)	
																					
M3	0	18	10	1.0		0.49	10	5	1.0		0.23	24	17	1.0		0.82	8	8	1.0		0.32
	1–4	5	16	0.2	(0.0, 0.8)		10	15	0.4	(0.1, 1.6)		8	16	0.2	(0.0, 0.9)		15	21	0.6	(0.2, 1.9)	
	⩾5	4	1	1.7	(0.2,19.3)		7	7	0.6	(0.1, 2.9)		1	0	—	—		10	4	2.0	(0.6, 7.3)	
																					
M4^e^	0	25	26	1.0		0.13	14	21	1.0		0.57	37	49	1.0		0.03	20	14	1.0		0.61
	1–4	23	26	0.8	(0.4, 1.8)		27	19	2.2	(0.8, 5.9)		32	23	1.9	(0.9, 4.1)		37	38	0.6	(0.3, 1.5)	
	⩾5	6	2	2.5	(0.5,13.2)		13	14	1.5	(0.5, 4.5)		4	1	4.5	(0.5,41.4)		16	21	0.5	(0.2, 1.5)	
																					
M5^f^	0	11	12	1.0		1.00	6	7	1.0		0.10	11	12	1.0		0.67	2	6	1.0		0.11
	1–4	6	7	0.5	(0.1, 2.7)		9	8	0.6	(0.1, 3.3)		8	9	0.8	(0.2, 2.7)		17	9	2.7	(0.3,27.0)	
	⩾5	2	0	—	—		4	4	0.3	(0.0, 3.2)		2	0	—	—		2	6	0.4	1(0.0,12.6)	

Abbreviations: Ca=cases; CI=confidence interval; Co=controls; FAB=French–American–British; OR=odds ratio; SES=socioeconomic status.

a Excludes pairs in which either the case or control had prior radiation therapy and/or chemotherapy for cancer.

b Cutoff between ‘high’ and ‘low’ dose to active bone marrow was 1 mGy.

c Actual number of procedures as a continuous variable.

d Includes 51 case-control pairs with unknown FAB subtype.

e Excludes cases with FAB subtype M4eo.

f Includes cases with FAB subtypes M5A and M5B.

**Table 4:** Risk analysis of total estimated bone marrow dose from diagnostic imaging procedures in the 2–10 years before diagnosis, adjusted for SES^a^

		**Data from medical records**					**Data from interview**				
**FAB group**	**Total estimated dose (mGy)^b^**	**# Ca**	**# Co**	**OR**	**(95% CI)**	***P* trend^c^**	**# Ca**	**# Co**	**OR**	**(95% CI)**	***P* trend^c^**
All^d^	⩽1000	234	236	1.0		0.28	295	290	1.0		0.70
	1001–2000	22	30	0.7	(0.4, 1.3)		31	46	0.6	(0.4, 1.0)	
	>2000	23	13	1.6	(0.8, 3.2)		18	8	2.5	(1.0, 6.0)	
											
M1	⩽1000	49	48	1.0		0.76	58	56	1.0		0.95
	1001–2000	4	3	1.3	(0.3, 5.9)		6	11	0.6	(0.2, 1.7)	
	>2000	2	4	0.5	(0.1, 2.9)		3	0	—		
											
M2	⩽1000	65	66	1.0		0.54	89	84	1.0		0.46
	1001–2000	6	10	0.7	(0.2, 2.1)		7	10	0.6	(0.2, 1.8)	
	>2000	8	3	2.2	(0.6, 9.0)		3	5	0.6	(0.1, 2.6)	
											
M3	⩽1000	22	24	1.0		0.27	27	31	1.0		0.18
	1001–2000	2	3	0.6	(0.1, 3.8)		1	2	0.5	(0.0, 5.5)	
	>2000	3	0	—			5	0	—	—	
											
M4^e^	⩽1000	39	48	1.0		0.10	57	64	1.0		0.054
	1001–2000	8	5	2.0	(0.5, 8.1)		12	8	1.4	(0.5, 4.0)	
	>2000	7	1	6.7	(0.8, 55.4)		4	1	4.7	(0.5,43.1)	
											
M5^f^	⩽1000	17	16	1.0		0.63	18	19	1.0		0.96
	1001–2000	0	2	0.0	(0.0, —)		1	2	0.6	(0.1, 7.7)	
	>2000	2	1	1.9	(0.1, 53.1)		2	0	—		
											

Abbreviations: Ca=cases; CI=confidence interval; Co=control; FAB=French-American-British; OR=odds ratio; SES=socioeconomic status.

a Excludes pairs in which either the case or control had prior radiation therapy and/or chemotherapy for cancer.

b Dose is likely to be consistently underestimated (see text).

c Total dose as a continuous variable.

d Includes 51 case-control pairs with unknown FAB subtype.

e Excludes cases with FAB subtype M4eo.

f Includes cases with FAB subtypes M5A and M5B.
