# Diagnostic radiography and adult acute myeloid leukemia: an interview and medical chart review study

**DOI:** 10.1038/bjc.2012.426

**Published:** 2012-09-25

**Authors:** J M Pogoda, P W Nichols, R K Ross, D O Stram, D C Thomas, S Preston-Martin

**Correction to**: British Journal of Cancer (2012) **104**, 1482–1486; doi:10.1038/bjc.2011.114

After publication, it was noted that the dose category labels contained in Table 4 of this paper were presented at magnitude 100 times higher than was correct.

The full, correct Table 4 is now presented, below:

## Figures and Tables

**Table 4:** Risk analysis of total estimated bone marrow dose from diagnostic imaging procedures in the 2–10 years before diagnosis, adjusted for SES^a^

		**Data from medical records**					**Data from interview**				
**FAB group**	**Total estimated dose (mGy)** b	**# Ca**	**# Co**	**OR**	**(95% CI)**	***P*** **trend**^c^	**# Ca**	**# Co**	**OR**	**(95% CI)**	***P*** **trend**^c^
All^d^	⩽10.00	234	236	1.0		0.28	295	290	1.0		0.70
	10.01–20.00	22	30	0.7	(0.4, 1.3)		31	46	0.6	(0.4, 1.0)	
	>20.00	23	13	1.6	(0.8, 3.2)		18	8	2.5	(1.0, 6.0)	
											
M1	⩽10.00	49	48	1.0		0.76	58	56	1.0		0.95
	10.01–20.00	4	3	1.3	(0.3, 5.9)		6	11	0.6	(0.2, 1.7)	
	>20.00	2	4	0.5	(0.1, 2.9)		3	0	—		
											
M2	⩽10.00	65	66	1.0		0.54	89	84	1.0		0.46
	10.01–20.00	6	10	0.7	(0.2, 2.1)		7	10	0.6	(0.2, 1.8)	
	>20.00	8	3	2.2	(0.6, 9.0)		3	5	0.6	(0.1, 2.6)	
											
M3	⩽10.00	22	24	1.0		0.27	27	31	1.0		0.18
	10.01–20.00	2	3	0.6	(0.1, 3.8)		1	2	0.5	(0.0, 5.5)	
	>20.00	3	0	—			5	0	—	—	
											
M4^e^	⩽10.00	39	48	1.0		0.10	57	64	1.0		0.054
	10.01–20.00	8	5	2.0	(0.5, 8.1)		12	8	1.4	(0.5, 4.0)	
	>20.00	7	1	6.7	(0.8, 55.4)		4	1	4.7	(0.5,43.1)	
											
M5^f^	⩽10.00	17	16	1.0		0.63	18	19	1.0		0.96
	10.01–20.00	0	2	0.0	(0.0, —)		1	2	0.6	(0.1, 7.7)	
	>20.00	2	1	1.9	(0.1, 53.1)		2	0	—		
											

Abbreviations: Ca=cases; CI=confidence interval; Co=control; FAB=French-American-British; OR=odds ratio; SES=socioeconomic status.

a Excludes pairs in which either the case or control had prior radiation therapy and/or chemotherapy for cancer.

b Dose is likely to be consistently underestimated (see text).

c Total dose as a continuous variable.

d Includes 51 case-control pairs with unknown FAB subtype.

e Excludes cases with FAB subtype M4eo.

f Includes cases with FAB subtypes M5A and M5B.

